# Effects of intraoperative individualized PEEP on postoperative atelectasis in obese patients: study protocol for a prospective randomized controlled trial

**DOI:** 10.1186/s13063-020-04565-y

**Published:** 2020-07-06

**Authors:** Chen Zhu, Jing-Wen Yao, Li-Xin An, Ya-Fan Bai, Wen-Jing Li

**Affiliations:** Department of Anesthesiology, Beijing Friendship Hospital, Capital Medical University, No. 95 Yongan Road, Xicheng District, Beijing, 100050 China

**Keywords:** Obesity, Cstat, Individualized PEEP, Atelectasis, Electrical impedance tomography

## Abstract

**Background:**

Obese patients undergoing general anesthesia and mechanical ventilation during laparoscopic abdominal surgery commonly have a higher incidence of postoperative pulmonary complications (PPCs), due to factors such as decreasing oxygen reserve, declining functional residual capacity, and reducing lung compliance. Pulmonary atelectasis caused by pneumoperitoneum and mechanical ventilation is further aggravated in obese patients. Recent studies demonstrated that individualized positive end-expiratory pressure (iPEEP) was one of effective lung-protective ventilation strategies. However, there is still no exact method to determine the best iPEEP, especially for obese patients. Here, we will use the best static lung compliance (Cstat) method to determine iPEEP, compared with regular PEEP, by observing the atelectasis area measured by electrical impedance tomography (EIT), and try to prove a better iPEEP setting method for obese patients.

**Methods:**

This study is a single-center, two-arm, prospective, randomized control trial. A total number of 80 obese patients with body mass index ≥ 32.5 kg/m^2^ scheduled for laparoscopic gastric volume reduction and at medium to high risk for PPCs will be enrolled. They will be randomly assigned to control group (PEEP5 group) and iPEEP group. A PEEP of 5 cmH_2_O will be used in PEEP5 group, whereas an individualized PEEP value determined by a Cstat-directed PEEP titration procedure will be applied in the iPEEP group. Standard lung-protective ventilation methods such as low tidal volumes (7 ml/kg, predicted body weight, PBW), a fraction of inspired oxygen ≥ 0.5, and recruitment maneuvers (RM) will be applied during and after operation in both groups. Primary endpoints will be postoperative atelectasis measured by chest electrical impedance tomography (EIT) and intraoperative oxygen index. Secondary endpoints will be serum IL-6, TNF-α, procalcitonin (PCT) kinetics during and after surgery, incidence of PPCs, organ dysfunction, length of in-hospital stay, and hospital expense.

**Discussion:**

Although there are several studies about the effect of iPEEP titration on perioperative PPCs in obese patients recently, the iPEEP setting method they used was complex and was not always feasible in routine clinical practice. This trial will assess a possible simple method to determine individualized optimal PEEP in obese patients and try to demonstrate that individualized PEEP with lung-protective ventilation methods is necessary for obese patients undergoing general surgery. The results of this trial will support anesthesiologist a feasible Cstat-directed PEEP titration method during anesthesia for obese patients in attempt to prevent PPCs.

**Trial registration:**

www.chictr.org.cn ChiCTR1900026466. Registered on 11 October 2019

## Background

It is quite certain that postoperative pulmonary complications (PPCs) result in more morbidity and mortality, as well as prolong hospital stays. According to the type of surgery and the definition of PPCs, the incidence of PPCs has been reported to range from 5 to 33% [1, 2]. Considering that approximately 234 million patients worldwide require surgical treatment under general anesthesia each year [3], reducing the incidence of PPCs may have a great impact on global mobility and mortality. In recent years, more and more attention is paid to intraoperative mechanical ventilation strategies, which may affect PPCs in addition to the preoperative optimization of patients’ status and operation style.

Recently, an international expert consensus recommendation about lung-protective ventilation for the surgical patient was reported [4]. In the expert consensus, the following was strongly recommended: preoperative pulmonary risk evaluation, an individualized mechanical ventilation which include a tidal volume (*V*_*T*_) of 6–8 ml/kg predicted body weight (PBW), positive end-expiratory pressure (PEEP) of 5 cmH_2_O, and alveolar recruitment maneuvers (RM). Among these lung-protective ventilation strategies, individualized PEEP is important to prevent processive alveolar collapse. RM can reverse alveolar collapse but have limited benefit without sufficient PEEP. However, how to set individualized PEEP remains a matter of debate.

Obese patients have a high risk of PPCs. In obese patients, lung function is impaired due to the reduction of oxygen reserve, functional residual capacity, and lung compliance. Especially in the general anesthesia of laparoscopic surgery, the formation of atelectasis caused by pneumoperitoneum and mechanical ventilation will be further aggravated, which will seriously affect the prognosis and outcome of obese patients [5–7]. In order to reduce the incidence of atelectasis, the PEEP level of obese patients should be much higher than that of non-obese patients [8, 9]. However, how to set up the ideal individualized PEEP for obese patients in laparoscopic surgery is still uncertain. In the past clinical practice, the PEEP value was often set at 5–10 cmH_2_O based on personal experience or according to the results of numerous studies applied to the optimal PEEP in non-obese patients. It was confirmed that the protective effect of generalized and empirical PEEP on lung function was much lower than that of individualized PEEP [9–11]. Nestler and colleagues [11] evaluated the effect of individualized PEEP titrated by electrical impedance imaging (EIT) in obese patients undergoing laparoscopic surgery. They found that compared with normal PEEP group, the iPEEP group reduced atelectasis, decreased ventilator driving pressure, and improved oxygenation in obese patients. And the iPEEP titrated by EIT was up to 18.5 cmH_2_O, which is far more than our routine used. Eichler [12] reported the results of using esophageal pressure measurement and EIT to adjust PEEP; they found an average iPEEP of 23.8 cmH_2_O was necessary. However, the iPEEP titration using electrical impedance imaging or esophageal pressure measurement is not always feasible in routine clinical practice [13]. Therefore, we need to find some simple methods of iPEEP titration with high clinical feasibility in obese patients.

It had been confirmed that there was a very clear correlation between the severity of inflammatory reaction and the concentration of serum procalcitonin (PCT) [14, 15]. Therefore, it is rational to believe that the inflammatory response can be monitored by regular PCT measurements during the perioperative period; hence, PCT kinetic monitoring can be used as an indicator of host inflammatory response.

This trial will verify the following hypothesis: individualized PEEP titrated by the best static lung compliance (Cstat), combined with other lung-protective ventilation strategies, compared with the conventional setting of PEEP, can reduce atelectasis and PPCs in obese patients at intermediate to severe risk for PPCs. Through evaluation of atelectasis and ventilation distribution by electrical impedance tomography (EIT) of obese patients before and after laparoscopic surgery, we confirm the effect of iPEEP on oxygenation and postoperative PPCs in obese patients. Meanwhile, we observe the changes of perioperative pulmonary inflammatory factors and other health indicators (length of stay, hospital costs), so as to confirm its effectiveness and significance of iPEEP in reducing postoperative pulmonary complications in obese patients.

## Methods/design

### Objectives and design

**Fig. 1:**
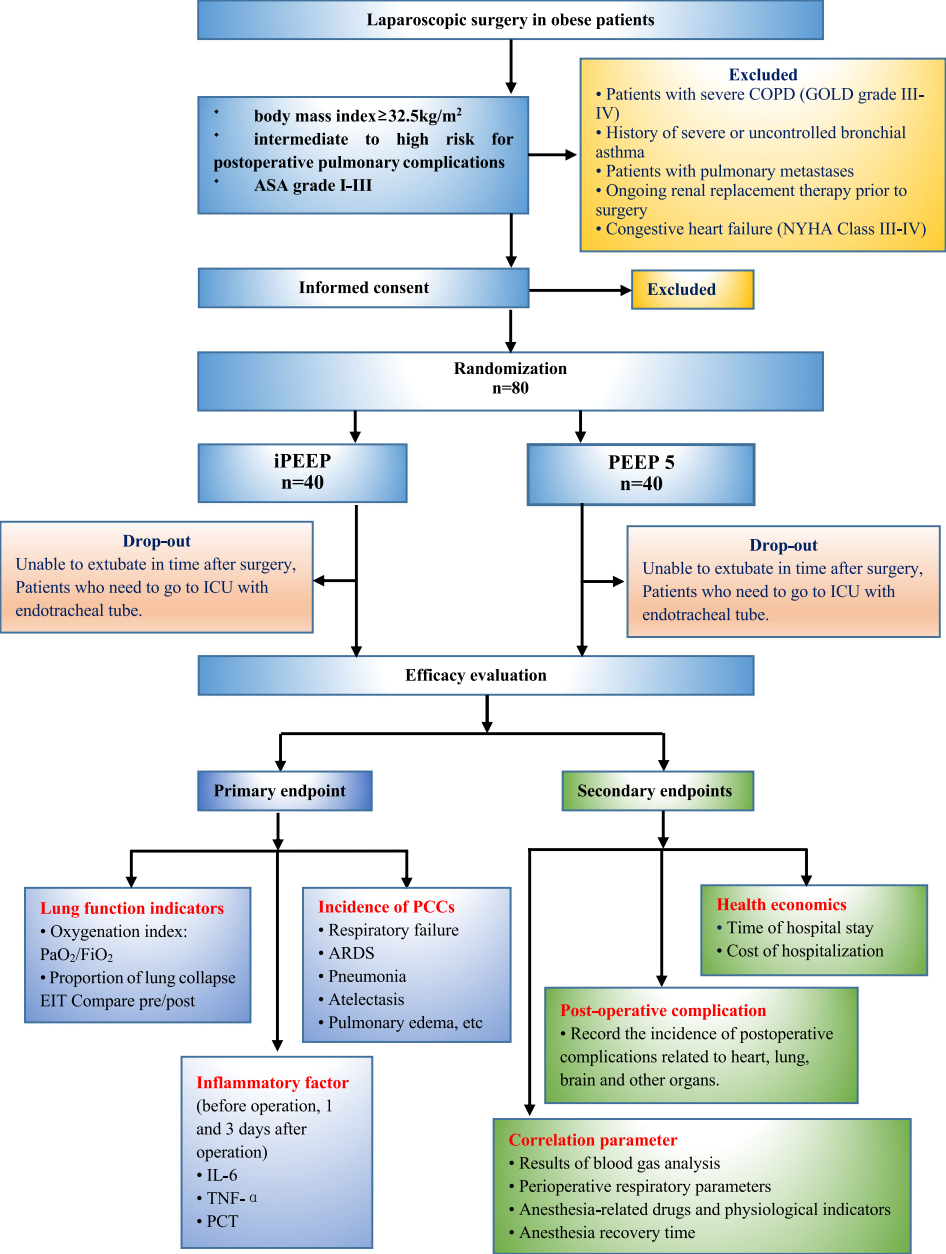
Consolidated Standards of Reporting Trials (CONSORT) diagram for this trail. PEEP, positive end-expiratory pressure; iPEEP, individual PEEP, COPD, chronic obstructive pulmonary disease, ICU, intensive care unit; ASA, American Society of Anesthesiologists classification, PaO_2_, partial pressure of arterial oxygen, FiO_2_, inspiratory fraction of inspired oxygen, EIT, electrical impedance tomography, PPCs, postoperative pulmonary complications, PCT, procalcitonin

This prospective, single-center, randomized, controlled, single-blind (patient-blinded, investigator-blinded) trial tests the hypothesis that individualized PEEP titrated by Cstat is an ideal lung-protective strategy for laparoscopic surgery in obese patients at intermediate and severe risk for PPCs. In total, 80 patients will be randomly assigned to one of two different intraoperative mechanical ventilation strategies (see Consolidated Standards of Reporting Trials [CONSORT] diagram, Fig. 1). The SPIRIT 2013 Checklist is given in Additional file 1.

This study will be conducted at the Beijing Friendship Hospital affiliated to Capital Medical University, China. The trial will be conducted according to the WMA of the Declaration of Helsinki and the CIOMS Principles of the International Guidelines for Biomedical Research Involving Human Subjects. This study has been approved by the Ethics Committee of the Beijing Friendship Hospital (the approval number from the Ethics Committee is 2019-P2-137-02) and has been registered in the Chinese Clinical Trial Registry (Chictr) (registration number: ChiCTR1900026466).

### Blinding, data collection, randomization, and record keeping

This is a single-blind study, participants are blinded, implementers are not blinded, and observers are blinded. Patients’ data, respiratory parameters, anesthesia data, fluid balance, laboratory results, postoperative clinical status, length of hospitality, and cost will be collected on case report forms (CRF).

All participants who meet the inclusion criteria are randomly divided into two groups, iPEEP group and PEEP5 group in a ratio of 1:1. Randomization will be performed by a computer-generated randomization table, with 20 blocks of four patients per block. Distribution will be stored in numbered, sealed, and opaque envelopes. Participants will be included and assigned in numerical order. All original records (informed consent, CRF, and related letters) will be archived and protected for 10 years, and then destroyed according to the hospital standards.

### Study population

Obese patients scheduled for laparoscopic metabolic and bariatric surgery will be screened and recruited during routine preoperative assessment. Participants meeting the inclusion criteria will be asked for signed informed consent. Inclusion criteria are as follows: BMI ≥ 32.5, 18–60 years old, American Society of Anesthesiologists (ASA) physical status I–III, and moderate or high risk for postoperative pulmonary complications. To identify patients at risk for PPCs, the Assess Respiratory Risk in Surgical Patients in Catalonia (ARISCAT) score is used [16]. This score predicts preoperative risk for PPCs using seven independent predictors, four of which are patient-related and three of which are surgery-related. An ARISCAT risk score ≥ 26 is associated with an intermediate to high risk for PPCs (Assess Respiratory Risk in Surgical Patients in Catalonia, ARISCAT ≥ 26) (Additional Fig 1). According to the Asia-Pacific classification, here, we used BMI ≥ 32.5 as the obese patient’s definition [17].

The exclusion criteria are as follows: patients aged < 18 years or > 60 years, ASA grade ≥ IV, severe chronic obstructive pulmonary disease (COPD, GOLD grades III–IV), a history of severe or uncontrolled bronchial asthma, patients with pulmonary metastases, ongoing renal replacement therapy before surgery, congestive heart failure (NYHA grades III–IV), and patients who cannot be extubated in time after surgery and need to return to the ICU with an endotracheal tube. For the specific clinical trial process, see Fig. 1.

### Standard procedures

In order to avoid interference with the trial intervention, perioperative anesthesia care (including induction and maintenance of general anesthesia, postoperative pain management, and fluid management) is performed by a relatively fixed anesthesia team according to clinical routine. The following approaches are suggested:

1. Adequate airway assessment is required in all patients, predicated on 12 predictors of a difficult airway, and when more than three predictors are present, consideration should be given to intubating the trachea with an awake endotracheal tube or with slow induction to preserve spontaneous breathing, establishing an airway and preparing adequate equipment, personnel, and drugs in advance (Attached Table 1: Predictors of Difficult Airway).
2. Patients are routinely monitored after admission in the operating room, such as blood pressure, electrocardiogram, pulse oxygen saturation, BIS, and urine volume. Invasive arterial pressure was monitored by radial or dorsalis pedis artery puncture under local anesthesia.
3. For patients without anticipated difficult airway, rapid sequence induction was used for anesthesia induction: midazolam 0.05 mg/kg is given 15 min before induction, anesthesia is induced with etomidate, sufentanil, rocuronium, or cisatracurium, and mechanical ventilation was performed after tracheal intubation.
4. Anesthesia is maintained with total intravenous anesthesia by using intravenous propofol and remifentanil.
5. The maintenance of intraoperative circulation is actively managed based on surgery procedure and bleeding.
6. Perform postoperative pain treatment to control a visual analogue scale (VAS) pain score < 3. Local incision anesthesia or neuraxial block should be performed.
7. Encourage early mobilization, deep breathing exercises, and stimulation of cough in the postoperative period.

Data on the procedures applied will be recorded in detail and analyzed. All anesthesia and related treatment need to comply with clinical routines. Nasogastric tubes and intravenous catheters may be used according to surgery practice or guidelines. Urinary bladder catheter is usually not inserted according to the routine for this type of surgery in our hospital.

### Mechanical ventilation

The breathing settings for mechanical ventilation are as follows: using the pressure-control-volume compensation mode (PSC-VC) to set the driving pressure to 15 cmH_2_O. We used the lowest possible fraction of inspired oxygen (FiO_2_ ≥ 0.5) to maintain a peripheral oxyhemoglobin saturation measured (SpO_2_) > 92%. The compensated tidal volume is set to 7 ml/kg (PBW) and the respiratory rate to 12–15 breaths/min (targeting P_ET_CO_2_ maintenance at 35–45 cmH_2_O). Anesthetic complications were managed according to clinical guidelines. Pulmonary ventilation was recorded by EIT observation before induction of anesthesia and after extubation in each group.

### Intervention

**Fig. 2:**
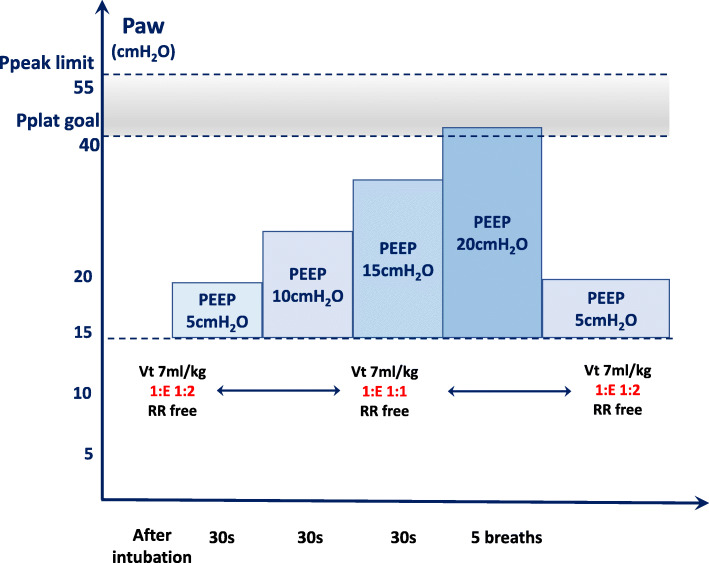
The ventilator-driven alveolar recruitment maneuver protocol. Ppeak, peak airway pressure; Pplat, plateau airway pressure; PEEP, positive end-expiratory pressure; *V*_*T*_, tidal volume normalized for adjusted body weight; I:E, ratio between inspiratory and expiratory time; RR, respiratory rate

After induction of anesthesia in all patients, PEEP was maintained at 5 cmH_2_O, mechanical ventilation was performed for 5 min, and baseline measurements of relevant parameters were performed in all patients. In the PEEP5 group, 5 cmH_2_O PEEP was maintained throughout the mechanical ventilation. Ventilator-driven alveolar recruitment maneuver (RM) is performed three times in both groups. In both groups, the first RM is performed at the moment of 5 min after intubation, the second RM is performed at the moment of establishment of pneumoperitoneum, and the third RM is at the moment before extubation. The ventilator-driven alveolar recruitment maneuver is performed as following steps [18] (Fig. 2):

1. In pressure control mode, the driving pressure is set to 15–20 cmH_2_O.
2. Starting at a PEEP of 5 cmH_2_O and increasing in steps of 5 cmH_2_O, each increase was maintained for 30 s until it was increased to a PEEP of 20 cmH_2_O, with a inspiratory plateau pressure as high as 40 cmH_2_O. Maintain 5 breaths at PEEP = 20 cmH_2_O to the end.
3. During the whole period of RM, *V*_*T*_ is 7 ml/kg, and I:E is 1:1.
4. Ppeak < 55 cmH_2_O.
5. A standardized fluid therapy regimen was used in all patients, and during RM, patients were given according to the protocol, along with vasopressors, to maintain MAP > 70 mmHg and minimize short-term hemodynamic suppression during RM.

**Fig. 3:**
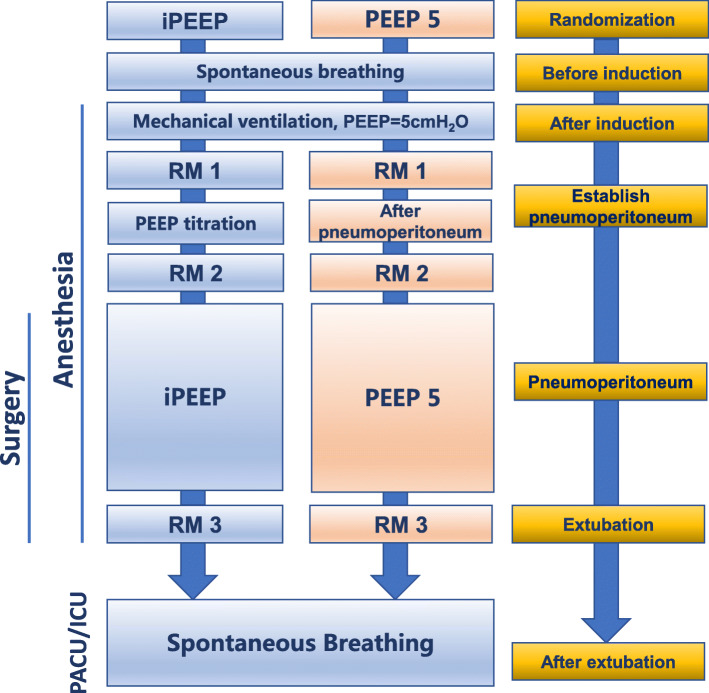
Individualized PEEP and perioperative management process. PEEP, positive end-expiratory pressure; iPEEP, individualized positive end-expiratory pressure; RM, the ventilator-driven alveolar recruitment maneuver; PEEP5, PEEP is 5 cmH_2_O

**Fig. 4:**
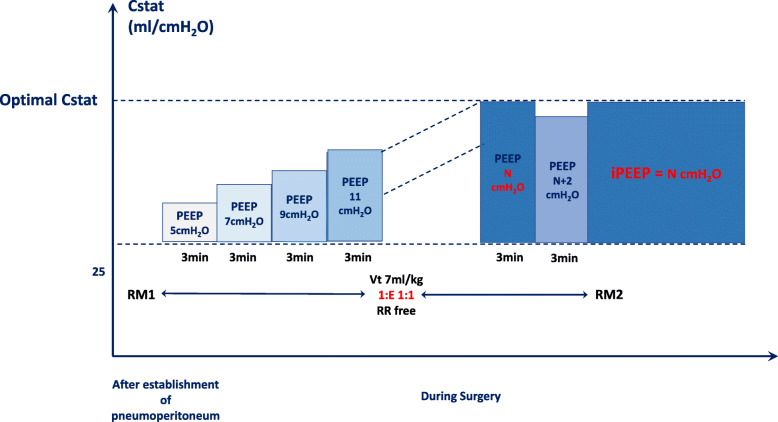
Individualized PEEP titrated by optimal Cstat. Cstat, static lung compliance; PEEP, positive end-expiratory pressure; iPEEP, individualized positive end-expiratory pressure; RM, the ventilator-driven alveolar recruitment maneuver; PEEP5, PEEP is 5 cmH_2_O

The iPEEP group obtained iPEEP for this patient by following these steps (Fig. 3):

1. First RM1: At 5 min after intubation, the first RM is performed.
2. Set airway peak pressure not to exceed 55 cmH_2_O.
3. *V*_*T*_ to 7 ml/kg (adjusted body weight, ABW), respiratory rate 12–15 breaths/min, I: E to 1:1.
4. Titration process: At the moment of establishment of pneumoperitoneum, we begin to titrate the iPEEP (Fig. 4). Setting initial PEEP to 5 cmH_2_O, increasing PEEP according to the gradient of 2 cmH_2_O every 3 min, calculating Cstat (according to the formula: Cstat = *V*_*T*_/Plat-PEEP). Gradually increasing PEEP, until the calculated Cstat shows a downward trend, set its previous PEEP (corresponding to PEEP for high Cstat) as the optimal iPEEP for this patient.
5. The highest PEEP is limited to 20 cmH_2_O.
6. After setting the iPEEP, the second RM2 is performed.
7. Before extubation, the third RM3 is performed.

### Study endpoints

The primary endpoints of this study are oxygenation index and proportion of lung collapse area.

1. Oxygenation index (PaO_2_/FiO_2_): At before induction (T0), after intubation (T1), after the last RM and before tracheal extubation (T3), and 20 min after extubation (T4), we draw arterial blood, perform blood gas analysis, and calculate oxygenation index.
2. Proportion of lung collapse area: After all patients were admitted to the operating room, the first lung volume and its related parameters were measured using Drager’s EIT instrument. Two hours after surgery, EIT was performed again to measure the proportion of the area occupied by the non-ventilated lung tissue and calculate the proportion of its lung collapse area.

Secondary clinical endpoints include the following: blood gas analysis indicators, respiratory parameters, anesthesia-related parameters, inflammatory factors, incidence of PCCs, and health economic indicators.

1. Blood gas analysis parameters: At before induction (T0), after intubation (T1), after the last RM and before tracheal extubation (T3), and 20 min after extubation (T4), we draw arterial blood and perform blood gas analysis.
2. Respiratory parameters: *V*_*T*_, RR, Pplat, PEEP, and Ppeak during operation are recorded every 5 min.
3. Inflammatory factors: 5 ml venous blood is taken before surgery, 1 day after surgery, and 3 days after surgery. The blood is centrifuged and frozen at once by professional clinical test staff in order to detect IL-6, TNF-α, and serum procalcitonin (PCT) concentration in future.
4. Anesthesia-related parameters: Circulatory parameters, anesthetic dosage, recovery time, and occurrence of hypoxemia are recorded continuously.
5. Postoperative follow-up-related respiratory function and health economic indicators: ICU stay, hospital stay, hospital costs, complications, and other adverse events were recorded.
6. PPCs is defined as following:
  1. Mild respiratory failure: PaO_2_ < 60 mmHg or SpO_2_ < 90%, effective for oxygen response of 2 L/min, except for low ventilation, at least 10 min under air inhalation.
  2. Moderate respiratory failure: PaO_2_ < 60 mmHg or SpO_2_ < 90%, effective only for oxygen response of > 2 L/min, except for low ventilation.
  3. Severe respiratory failure: requiring support of mechanical ventilation or invasive ventilation.
  4. ARDS: ARDS according to Berlin definition.
  5. Bronchospasm (newly detected expiratory wheezing treated with bronchodilators).
  6. New pulmonary infiltrative inflammation (confirmed by chest X-ray, but no other clinical signs).
  7. Pulmonary infection (new or progressive radiographic infiltrate plus at least two of the following: antibiotic treatment, tympanic temperature > 38 °C, leukocytosis or leukopenia [white blood cell count < 4000 cells/mm^3^ or > 12,000 cells/mm^3^], and/or purulent secretions).
  8. Aspiration pneumonitis (respiratory failure after the inhalation of regurgitated gastric contents).
  9. Pleural effusion (chest X-ray demonstrating blunting of the costophrenic angle, loss of the sharp silhouette of the ipsilateral hemidiaphragm in upright position, evidence of displacement of adjacent anatomical structures, or [in supine position] a hazy opacity in one hemithorax with preserved vascular shadows).
  10. Atelectasis (lung opacification with shift of the mediastinum, hilum, or hemidiaphragm toward the affected area, as well as compensatory overinflation in the adjacent nonatelectatic lung).
  11. Cardiopulmonary edema (clinical signs of congestion, including dyspnea, edema, rales, and jugular venous distention, with chest X-ray demonstrating increase in vascular markings and diffuse alveolar interstitial infiltrates).
  12. Pneumothorax (air in the pleural space with no vascular bed surrounding the visceral pleura).

One of the above conditions was defined as positive for PCCs.

### Study visits and data collection

**Fig. 5:**
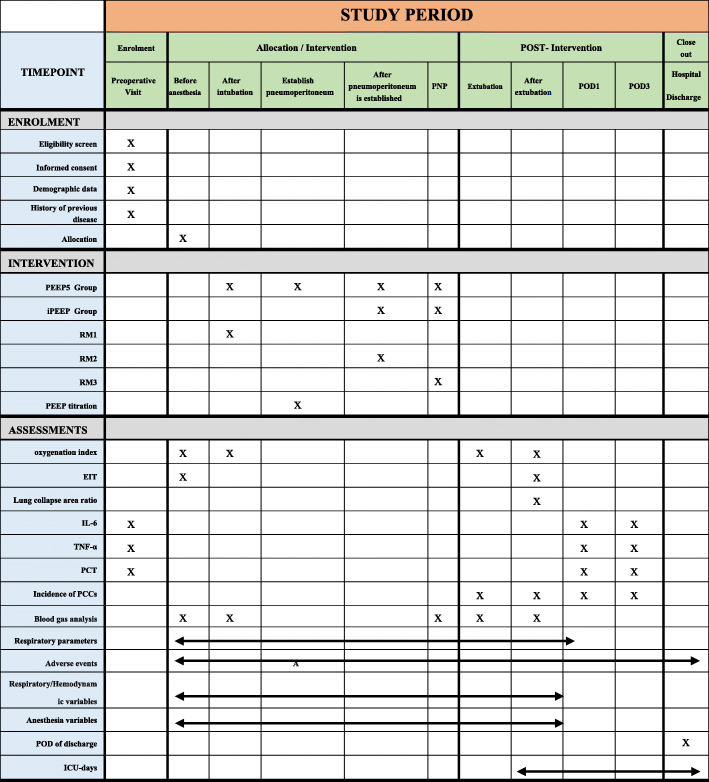
Standard protocol items: time schedule of enrollment, interventions, and assessments. PEEP, positive end-expiratory airway pressure; iPEEP, individual PEEP; RM, recruitment maneuver; EIT, electrical impedance tomography; PNP, pneumoperitoneum; PCT, serum procalcitonin; PPCs, postoperative pulmonary complications; POD, postoperative day; ICU, intensive care unit

The patients are followed up preoperatively, intraoperatively, 1 day after operation, and 3 days after surgery and at discharge (Fig. 5). At different stages, patient’s data is collected and recorded:

1. Preoperative indicators: age, gender, height, weight, BMI, smoking history, difficult airway assessment, ASA grade, blood pressure, heart rate, body temperature, blood routine, biochemical items, coagulation function, other past disease history.
2. Intraoperative indicators: blood pressure, heart rate, pulse oxygen saturation, BIS value, respiratory parameters, infusion volume, blood transfusion volume, urine volume, analgesic drug dose, sedative drug dose, muscle relaxant drug dose, vasoactive drug dose, operation time, blood gas analyze, etc.
3. Postoperative indicators: ICU treatment time, hospital stay, hospital costs, any complications and adverse events.

### Study dropouts

Since participation in the trial is voluntary, subjects have the right to withdraw their consent to participate in the study at any time and for any reason without any further treatment. In addition, if the investigator believes that the participation of any subject is in the best interests of the subject, the investigator has the right to terminate his participation at any time. The reasons and circumstances for stopping the study will be documented in the CRF.

### Sample size calculations

The primary outcome measures of this study were oxygenation index (PaO_2_/FiO_2_) and the ratio of lung collapse area to normal lung tissue by preoperative and postoperative EIT. According to the literature report [10], the ratio of pulmonary collapse detected by EIT in obese patients with individualized peep (iPEEP) set by EIT compared with PEEP 5 cmH_2_O was 6.2 ± 4.1% vs. 10.8 ± 7.1%, with a significant difference (*P* = 0.017). We hypothesize that the Cstat-titrated iPEEP will improve the rate of postoperative lung collapse in obese patients, with a probability of *α* = 0.05 to allow for type 1 error, *β* = 0.1 to allow type 2 error, and power 0.90; according to the mean of two groups obtained in the literature, 35 cases are needed for each group by using the PASS 20.0 software. A final sample size of 40 per group accounts for a 10% dropout rate in case follow-up.

### Data monitoring

This study is composed of a principal investigator, general investigator, and participants who contributed to the design and practice of the study protocol, participated in the experimental process of this study, and recorded the experimental data. The data monitoring for this study will be performed centrally by an external independent physician who will not be involved in the study for quality control purposes. Monitoring will assess the progress of the study and verify the accuracy and completeness of data recording. At the end of the study, the original data and results will be submitted to the scientific research management committee, and they will be disclosed to the public after the results are published.

### Statistical analysis

After the trial, the research team will work with medical statisticians to analyze the data. Statistical analysis will be based on intention to treat. SPSS 20.0 statistical software was used for analysis.

The most of the source data will be recorded onto the CRF; however, before data analyzing, the pattern of missing data will be evaluated. The analysis of the graphics and data obtained by the EIT machine will be conducted by a special computer expert familiar with machine principles.

All data distribution will be detected by Kolmogorov-Smirnov analysis. Normal distribution data will be represented by mean and standard deviation (SD), and skewed data will be represented by median (quartile range). Compared with the related samples, *t* test will be used for normal distribution data; Wilcoxon signed-rank test and Mann-Whitney *U* test will be used for skewed data. The difference in proportions will be evaluated using Fisher’s exact test and the risk ratio of the associated 95% confidence interval (CI). The data of VAS score, ICU days, hospitalization days, and hospitalization costs will be analyzed by chi-square test. *P* value < 0.05 will be considered significant.

## Discussion

This study is adequately powered to test the hypothesis that an individualized PEEP titrated by Cstat ventilation strategy can benefit obese patients in terms of perioperative oxygenation index, proportion of collapsed lung area, change in inflammatory factors, and incidence of PCCs, compared with an ordinary PEEP ventilation strategy.

PPCs related to general anesthesia and mechanical ventilation have attached more and more attention. Evidence suggests that lung-protective ventilation strategies are found to be effect on reducing the incidence of PPCs. Young and other six experts [4] reached an international expert consensus which included recommendations such as low *V*_*T*_, PEEP, and ARM. And in this consensus, an individualized PEEP was strongly emphasized. But how to set an iPEEP level, especially for special obese patients, is still uncertain.

Obesity has become a global health problem. During 2013–2016, 38.9% of US adults had obesity and 7.6% had severe obesity [19]. In obese patients, the accumulation of fat in the chest and abdomen limits thoracic activities. In supine position, the abdominal organs push up the diaphragm, further limiting the lung-thoracic compliance. In addition, the alveolar ventilation volume is decreased and FRC is reduced in these patients. Pulmonary infection and atelectasis are easy to occur during mechanical ventilation under general anesthesia [8–12]. Pneumoperitoneum pressure during laparoscopic surgery further reduces chest wall and lung compliance in obese patients and significantly increases the probability of intraoperative hypoxia and PPCs. The incidence of atelectasis after upper abdominal surgery in obese patients is as high as 45% and can last several weeks after surgery. For patients undergoing general anesthesia with mechanical ventilation, about 75% of patients develop a state of local alveolar non-ventilation during surgery. Local atelectasis leads to ventilation/blood flow imbalance and intrapulmonary shunt and even induce hypoxemia, which is more pronounced in obese patients [20–23]. Compared with zero end-expiratory pressure (ZEEP), PEEP can improve end-expiratory long volume (EELV), increase oxygenation, and improve respiratory system compliance (C_RS_), dependent lung ventilation, and post-operative lung function [24]. However Pirrone et al [25] found that PEEP commonly used by clinicians was insufficient for mechanical ventilation in morbidly obese patients.

Low tidal volume is one of the lung-protective ventilation strategies. Although low tidal volume can significantly reduce the incidence of ventilator-associated lung injury and reduce mortality, low tidal volume ventilation is not conducive to recruitment of collapsed alveoli in obese patients [13, 26]. Alveolar recruitment maneuvers (ARMs) are beneficial in reopening collapsed alveoli. After recruitment of collapsed alveoli, appropriate PEEP needs to be selected to maintain and prevent alveolar collapse again. However, too high PEEP will lead to alveolar overdistension and aggravate lung injury, while too low PEEP will lead to alveolar collapse again. Therefore, finding the optimal PEEP level is a problem that has been continuously explored in clinical practice.

The earliest goal of PEEP was to correct hypoxia, and many physiological studies have shown that PEEP levels of at least 5 cmH_2_O are necessary. Most scholars recommend that obese patients should be given 10 cmH_2_O PEEP, but the correction of hypoxia is not the ultimate goal. A reasonable PEEP should improve hypoxia, promote recruitment of collapsed alveoli, and prevent overdistension of alveoli [27]. Relevant studies have shown that individualized PEEP strategy compared with ordinary PEEP ventilation strategy can counteract the reduction of end-expiratory volume, improve respiratory mechanics and reduce intrapulmonary shunt, enhance oxygenation capacity, and improve the patient’s intraoperative ventilation [6].

How to set an ideal and individualized PEEP for obese patients in laparoscopic surgery is a difficult problem. The setting of PEEP shall be individualized to eliminate the effect of chest wall and abdominal high pressure on actual transpulmonary pressure and effectively improve the heterogeneity of gas distribution in the lungs. In this study, by gradually increasing and maintaining PEEP at 5 to 20 cmH_2_O, increasing the PEEP step by step according to the gradient until the calculated Cstat shows a decreasing trend; their previous PEEP (PEEP corresponding to the highest Cstat) will be set as the optimal iPEEP for this obese patient. According to our clinical work habits and other research recommendations [10, 11, 14], we set 5 cmH_2_O PEEP value as our control group PEEP, so as to verify the effectiveness of Cstat titration iPEEP.

Chest CT can accurately evaluate the size of lung recruitment and PEEP-induced lung recruitment. It calculates the tissue volume and gas volume in the lung tissue region by measuring the CT value. However, it is difficult to perform clinically and has the disadvantages of ionizing radiation. Electrical impedance tomography (EIT) can provide the gas distribution characteristics and mechanical characteristics of local lung tissues of patients by monitoring the change of intrathoracic impedance from lung ventilation [24, 28]. Compared with gold standard CT, EIT has the advantages of non-invasive, bedside, real-time, no radiation, etc. It has gradually become a research hotspot of clinical application and lung-protective ventilation in obese patients. EIT allows visual comparison and evaluation of regional lung tissue collapse and recruitment, as well as determination of lung volume and correlation with global respiratory mechanics. The application of EIT makes it possible to detect the patient’s lung collapse at the bedside.

In this study, ventilator-driven alveolar lung recruitment will be performed using the incremental PEEP method with the target PEEP set at 20 cmH_2_O and the target Ppeak set at 40 cmH_2_O. Individualized PEEP will be obtained by Cstat titration in obese patients. The effect of iPEEP on perioperative oxygenation index, lung collapse area, and incidence of PPCs in obese patients will be observed. At the same time, through detection of inflammatory factors in perioperative period, we tried to explore the mechanism of iPEEP on lung-protective function in obese patients.

In conclusion, this study tried to verify the following hypotheses: individualized PEEP titrated by Cstat combined with other lung-protective strategies will improve oxygenation and decrease atelectasis in obese patients during laparoscopic surgery. This study will provide a simple and feasible individualized PEEP titration method in obese patients. The result will provide direct clinical evidence for the next step to further refine the specific implementation of lung protection strategies in obese patients and add them to the link of surgical ERAS in obese patients.

## Trial status

The first participant was enrolled on November 3, 2019, and the first version was developed on June 26, 2019, the protocol version is the first version and the No is V1.0/2019.06.26. The recruitment will be completed on December 31, 2020. To date, 36 participants have been recruited. This trial is still ongoing.

## Supplementary information

**Additional file 1.** SPIRIT 2013 Checklist: Recommended items to address in a clinical trial protocol and related documents.

**Additional file 2: Fig 1.** Independent predictors of risk for development of postoperative pulmonary complications as described by Canet et al. ^[1]^ (ARISCAT score). A risk score ≥ 26 predicts an intermediate to high risk for postoperative pulmonary complications). ^a^ The simplified risk score is the sum of each logistic regression coefficient multiplied by 10, after rounding off its value. **Table 1.** Difficult Mask Ventilation Combined with Difficult Laryngoscopy Prediction Score ^[2]^.

## Data Availability

After the study is completed, the data will be open to the public through the Research Manager (ResMan) platform (http://www.medresman.org/login.aspx) within 6 months.

## Abbreviations

PPCs: Postoperative pulmonary complications
iPEEP: Individualized positive end-expiratory pressure
Cstat: Static lung compliance
FiO_2_: Inspiratory fraction of inspired oxygen
EIT: Electrical impedance tomography
PBW: Predicted body weight
RM: Recruitment maneuvers
*V*_*T*_: Tidal volume
CRF: Case report forms
ARISCAT: The Assess Respiratory Risk in Surgical Patients in Catalonia
BMI: Body mass index
COPD: Chronic obstructive pulmonary disease
VAS: Visual analogue scale
PSC-VC: Pressure-control-volume compensation mode
Pplat: Plateau airway pressure
Ppeak: Peak airway pressure
1:E: Ratio between inspiratory and expiratory time
PaO_2_: Partial pressure of arterial oxygen
PCT: Procalcitonin

## References

[1] Canet J, Gallart L, Gomar C (2010). Prediction of postoperative pulmonary complications in a population-based surgical cohort. Anesthesiology.

[2] Fernandez-Bustamante A, Frendl G, Sprung J (2017). Postoperative pulmonary complications, early mortality, and hospital stay following noncardiothoracic surgery: a multicenter study by the perioperative research network investigators. JAMA Surg.

[3] Weiser TG, Makary MA, Haynes AB, Dziekan G, Berry WR, Gawande AA (2009). Standardised metrics for global surgical surveillance. Lancet.

[4] Young CC, Harris EM, Vacchiano C (2019). Lung-protective ventilation for the surgical patient: international expert panel-basedconsensus recommendations. Br J Anaesth.

[5] Mazo V, Sabate S, Canet J (2014). Prospective external validation of a predictive score for postoperative pulmonary complications. Anesthesiology.

[6] Serpa Neto A, Hemmes SN, Barbas CS, Beiderlinden M, Fernandez-Bustamante A, Futier E (2014). Incidence of mortality and morbidity related to postoperative lung injury in patients who have undergone abdominal or thoracic surgery: a systematic review and meta-analysis. Lancet Respir Med.

[7] Canet J, Sabaté S, Mazo V, Gallart L (2015). Development and validation of a score to predict postoperative respiratory failure in a multicentre European cohort: a prospective, observational study. Eur J Anaesthesiol.

[8] Ball L, Hemmes SNT, Serpa Neto A (2018). Intraoperative ventilation settings and their associations with postoperative pulmonary complications in obese patients. Br J Anaesth.

[9] Pelosi P, Gregoretti C (2010). Perioperative management of obese patients. Best Pract Res Clin Anaesthesiol.

[10] Pereira SM, Tucci MR, Morais CCA (2018). Individual positive end-expiratory pressure settings optimize intraoperative mechanical ventilation and reduced postoperative atelectasis. Anesthesiology.

[11] Nestler C, Simon P, Petroff D (2017). Individualized positive end-expiratory pressure in obese patients during general anaesthesia: a randomized controlled clinical trial using electrical impedance tomography. Br J Anaesth.

[12] Eichler L, Truskowska K, Dupree A (2018). Intraoperative ventilation of morbidly obese patients guided by transpulmonary pressure. Obes Surg.

[13] Carron M (2018). Positive end-expiratory pressure in obese patients during general anaesthesia. The role of intra-abdominal pressure. Br J Anaesth.

[14] Ruszkai Z, Kiss E, László I (2017). Effects of intraoperative PEEP optimization on postoperative pulmonary complications and the inflammatory response: study protocol for a randomized controlled trial. Trials.

[15] Minami E, Ito S, Sugiura T (2014). Markedly elevated procalcitonin in early postoperative period in pediatric open heart surgery: a prospective cohort study. J Intensive Care.

[16] Bluth T, Teichmann R, Kiss T (2017). Protective intraoperative ventilation with higher versus lower levels of positive end-expiratory pressure in obese patients (PROBESE): study protocol for a randomized controlled trial. Trials.

[17] Lim JU, Lee JH, Kim JS, Hwang YI, Kim TH, Lim SY, Yoo KH, Jung KS, Kim YK, Rhee CK (2017). Comparison of World Health Organization and Asia-Pacific body mass index classifications in COPD patients. Int J Chron Obstruct Pulmon Dis.

[18] Güldner A, Kiss T, Serpa Neto A, Hemmes SN, Canet J, Spieth PM, Rocco PR, Schultz MJ, Pelosi P, Gama de Abreu M (2015). Intraoperative protective mechanical ventilation for prevention of postoperative pulmonary complications: a comprehensive review of the role of tidal volume, positive end-expiratory pressure, and lung recruitment maneuvers. Anesthesiology.

[19] Hales CM, Fryar CD, Carroll MD (2018). Differences in obesity prevalence by demographic characteristics and urbanization level among adults in the United States, 2013-2016. JAMA.

[20] Littleton SW, Tulaimat A (2017). The effects of obesity on lung volumes and oxygenation. Respir Med.

[21] Dixon AE, Peters U (2018). The effect of obesity on lung function. Expert Rev Respir Med.

[22] Zewari S, Vos P, van den Elshout F (2017). Obesity in COPD: revealed and unrevealed issues. COPD.

[23] Hedenstierna G, Tokics L, Scaramuzzo G (2019). Oxygenation impairment during anesthesia: influence of age and body weight. Anesthesiology.

[24] Pfurtscheller K, Ring S, Beran E (2015). Effect of body position on ventilation distribution during PEEP titration in a porcine model of acute lung injury using advanced respiratory monitoring and electrical impedance tomography. Intensive Care Med Exp.

[25] Pirrone M, Fisher D, Chipman D (2016). Recruitment maneuvers and positive end-expiratory pressure titration in morbidly obese ICU patients. Crit Care Med.

[26] De Jong A, Chanques G, Jaber S (2017). Mechanical ventilation in obese ICU patients: from intubation to extubation. Crit Care.

[27] Xie J, Jin F, Pan C (2017). The effects of low tidal ventilation on lung strain correlate with pulmonary compliance. Crit Care.

[28] He X, Jiang J, Liu Y (2016). Electrical impedance tomography-guided PEEP titration in patients undergoing laparoseopic abdominal surgery. Medicine (Baltimore).

